# Mapping the Scaffolding of Metacognition and Learning by AI Tools in STEM Classrooms: A Bibliometric–Systematic Review Approach (2005–2025)

**DOI:** 10.3390/jintelligence13110148

**Published:** 2025-11-15

**Authors:** Maria Tsakeni, Stephen C. Nwafor, Moeketsi Mosia, Felix O. Egara

**Affiliations:** Department of Mathematics, Natural Sciences and Technology Education, Faculty of Education, University of the Free State, Bloemfontein 9301, South Africa; tsakenim@ufs.ac.za (M.T.); nwafor.sc@ufs.ac.za (S.C.N.); mosiams@ufs.ac.za (M.M.)

**Keywords:** artificial intelligence, educational technology, metacognition, intelligent tutoring systems, posthumanism, STEM education

## Abstract

This study comprehensively analyses how AI tools scaffold and share metacognitive processes, thereby facilitating students’ learning in STEM classrooms through a mixed-method research synthesis combining bibliometric analysis and systematic review. Using a convergent parallel mixed-methods design, the study draws on 135 peer-reviewed articles published between 2005 and 2025 to map publication trends, author and journal productivity, keyword patterns, and theoretical frameworks. Data were retrieved from Scopus and Web of Science using structured Boolean searches and analysed using Biblioshiny and VOSviewer. Guided by PRISMA 2020 protocols, 24 studies were selected for in-depth qualitative review. Findings show that while most research remains grounded in human-centred conceptualisations of metacognition, there are emerging indications of posthumanist framings, where AI systems are positioned as co-regulators of learning. Tools like learning analytics, intelligent tutoring systems, and generative AI platforms have shifted the discourse from individual reflection to system-level regulation and distributed cognition. The study is anchored in Flavell’s theory of metacognition, General Systems Theory, and posthumanist perspectives to interpret this evolution. Educational implications highlight the need to reconceptualise pedagogical roles, integrate AI literacy in teacher preparation, and prioritise ethical, reflective AI design. The review provides a structured synthesis of theoretical, empirical, and conceptual trends, offering insights into how human–machine collaboration is reshaping learning by scaffolding and co-regulating students’ metacognitive development in STEM education.

## 1. Introduction

The 21st century has seen a profound transformation in education through adaptive teaching, digital technologies, and artificial intelligence (AI), especially in STEM (Science, Technology, Engineering, and Mathematics) subjects (Egara et al. 2025; Mosia and Egara 2025). As STEM education shifts toward personalised and reflective learning, metacognition—learners’ ability to monitor, control, and direct their thinking—has become increasingly critical (Stanton et al. 2021). Building on Flavell’s (1979) foundational work, metacognition is understood to involve both knowledge of one’s cognitive processes and the regulation of strategies such as planning, monitoring, and evaluation (Schraw and Moshman 1995).

Metacognition is particularly important in STEM, where problem-solving and experimentation dominate over rote learning (Broadbent and Poon 2015; Chen et al. 2021; Utami et al. 2023). Numerous studies link metacognitive awareness to improved conceptual understanding, motivation, reasoning, and academic performance (Berthold et al. 2011; Taran and Nalla 2019; Jiang et al. 2023; Tak et al. 2025). Interventions such as mentoring and structured reflection further enhance self-regulation and comprehension in technology-rich environments (Azevedo and Cromley 2004). However, fostering metacognition remains challenging due to limited teacher training, time, and resources, as well as the difficulty of observing internal cognitive processes (Margot and Kettler 2019; Gamby and Bauer 2022). These challenges underscore the need for innovative approaches—such as AI-powered tools—to support metacognitive development in STEM classrooms.

Recent advancements in AI offer promising avenues to address these challenges. AI-powered tools, such as Intelligent Tutoring Systems (ITS), adaptive platforms, learning analytics dashboards, and conversational agents, can scaffold learners’ metacognitive development by providing personalised feedback, real-time monitoring, and strategic prompts (Bond et al. 2024; Desmarais and Baker 2012; Egara and Mosimege 2024a, 2024b, 2024c). For example, platforms such as Carnegie Learning and ALEKS promote both content mastery (object level) and reflective monitoring (meta level) (Albus and Seufert 2025). Adaptive AI approaches, including deep reinforcement learning, have also shown potential to enhance metacognitive outcomes (Abdelshiheed et al. 2023). Bibliometric evidence further indicates a growing focus on ITS and related technologies that enable teachers to observe and support metacognitive behaviours (Cuéllar-Rojas et al. 2022).

However, ethical concerns remain if AI prioritises automation and speed over reflection, prompting scholars to call for responsible, transparent, and learner-centred AI integration (Lodhi 2025; Wang et al. 2024; Smith-Mutegi et al. 2025). Given both the promise and pitfalls of AI in supporting metacognition, it is crucial to understand how this intersection has evolved. While numerous empirical studies have examined individual AI tools or specific metacognitive strategies, existing reviews tend to focus broadly on AI in education without explicitly addressing how AI supports metacognition within STEM classrooms (Cuéllar-Rojas et al. 2022; Fatimah et al. 2024). However, these reviews rarely integrate bibliometric mapping with systematic qualitative synthesis, leaving gaps in understanding publication trends, theoretical framings, and the evolution of concepts over time. This study addresses these gaps by combining bibliometric analysis of 135 publications (2005–2025) with a systematic review of 24 core studies, offering a comprehensive two-decade overview of how AI tools scaffold and co-regulate metacognition in STEM education. By doing so, it provides valuable insights for researchers, educators, and policymakers seeking to design effective, ethically grounded AI-supported learning environments.

### 1.1. Theoretical Framework

This study is guided by three complementary frameworks: Flavell’s Theory of Metacognition (1979), General Systems Theory (GST), and Human-Centred vs. Posthumanist AI paradigms. Together, they provide a multidimensional lens for understanding how AI tools scaffold metacognition in STEM education.

Rather than redefining metacognition, we apply Flavell’s model (as introduced above) to interpret how AI tools support regulation at both the object level (task performance) and the meta level (strategic monitoring), following Nelson and Narens’ (1990) distinction (Schraw and Moshman 1995; Albus and Seufert 2025). Nelson and Narens’ (1990) distinction between the *object level* (task performance) and *meta level* (strategic regulation) is integrated here to illustrate how AI operates simultaneously in content mastery and reflective monitoring.

GST conceptualises STEM classrooms as interconnected, adaptive systems where students, teachers, technologies, and pedagogies interact dynamically (Von Bertalanffy 1950, 1968; Mitchell 2009). This perspective helps map how AI tools interact with cognitive and instructional subsystems, creating system-level effects that can be traced across the literature.

Ultimately, the human-centred paradigm regards AI as a means of supporting human agency under transparent and ethical oversight (Shneiderman 2020; Atchley et al. 2024). In contrast, the posthumanist paradigm challenges human–machine binaries by conceptualising learning as a co-regulated process distributed between humans and AI systems (Cukurova 2024; Lim 2025). We use these paradigms to interpret whether AI is framed as a supportive tool (human-centred) or a co-agent in reflective processes (posthumanist).

### 1.2. Literature Review

This section reviews key studies on metacognition in STEM education and the role of AI in supporting metacognitive processes. It highlights evidence of improved learning outcomes through the use of metacognitive strategies. It examines how AI tools have been integrated to enhance self-regulation, reflection, and critical thinking in STEM contexts.

#### 1.2.1. Metacognition and STEM Education

Metacognition—the ability to plan, monitor, and evaluate one’s thinking—is critical for problem-solving and conceptual understanding in STEM contexts (Flavell 1979; Tanner 2017). Studies consistently show its positive impact on academic performance across disciplines, including mathematics, science, chemistry, and physics (Smith and Mancy 2018; Zepeda et al. 2019; Adadan 2020; González et al. 2017; Yang 2012).

Research also links metacognitive skills to motivation and reasoning (Taran and Nalla 2019; Jiang et al. 2023; Tak et al. 2025), highlighting benefits for low-achieving and underrepresented groups (Ben-David and Zohar 2009; Rajcoomar et al. 2025; Clark et al. 2025). Despite this, challenges persist due to inconsistent measurement methods (Akturk and Sahin 2011), theoretical fragmentation (Azevedo 2020), and variability across contexts (Clary et al. 2018; Lavi et al. 2019). These gaps underscore the need for integrated theoretical approaches (Zohar and Barzilai 2013), which align with the objectives of the present review.

#### 1.2.2. Artificial Intelligence and Metacognition in STEM Education

AI systems—ranging from intelligent tutoring systems and adaptive platforms to generative AI tools like ChatGPT (versions GPT-4.1, GPT-4.5 and GPT-5)—are increasingly used to scaffold metacognition by providing real-time feedback and strategic prompts (Desmarais and Baker 2012; El Fathi et al. 2025). Learning analytics dashboards and conversational agents help externalise metacognitive processes by tracking learner progress and supporting reflection (Roll and Winne 2015; Holstein and Aleven 2021; Albus and Seufert 2025).

Recent studies demonstrate AI’s role in enhancing self-regulation, motivation, and retention through metacognitive prompts, chatbot feedback, and ME-CoT approaches (Markandan et al. 2022; Yin et al. 2024; Clark et al. 2025). Furthermore, AI has been linked to computational and critical thinking skills, fostering deeper cognitive engagement (Zhang et al. 2024; Lim 2025). However, concerns remain regarding cognitive overload, reduced learner autonomy, and algorithmic bias if AI prioritises automation over deep reflection (Tankelevitch et al. 2023; Chardonnens 2025; Levin et al. 2025).

While several bibliometric reviews have examined AI in education broadly, none focus explicitly on the intersection of AI and metacognition in STEM education (Cuéllar-Rojas et al. 2022; Fatimah et al. 2024). This study addresses that gap by mapping publication trends, theoretical frameworks, and conceptual shifts, with attention to the emerging discourse moving from human-centred approaches to posthumanist framings. The following research questions guide the review:

1. What are the trends in publication on metacognition in STEM education between 2005 and 2025?
2. What are the most frequently occurring AI-related concepts and tools in the literature on metacognition in STEM education?
3. Which journals, authors, and countries contribute most to the literature on AI and metacognition in STEM education?
4. Which theoretical frameworks are most frequently associated with studies on AI and metacognition in STEM, and how have these evolved?
5. How have keywords and conceptual language in the literature shifted from human-centred to posthumanist paradigms in the context of AI and metacognition?

## 2. Materials and Methods

This study adopted a convergent parallel mixed-method research synthesis design, combining bibliometric analysis with a systematic review. Both components were conducted independently but addressed complementary aspects of the research questions. The bibliometric analysis provided a quantitative map of trends, conceptual clusters, and publication networks spanning the period from 2005 to 2025. In parallel, the systematic review offered qualitative insights into how theoretical frameworks (including posthumanist paradigms) were employed in studies that integrated AI tools with metacognitive strategies in STEM classrooms. Findings from both strands were integrated during interpretation, allowing for cross-validation and thematic synthesis of patterns emerging from both data sources.

### 2.1. Data Sources and Search Strategy

Searches were conducted in Scopus and Web of Science, two multidisciplinary databases recognised for their credibility and extensive coverage of peer-reviewed research (Mongeon and Paul-Hus 2016). These databases were selected to ensure a comprehensive and diverse capture of studies at the intersection of AI, metacognition, and STEM education. Boolean operators and structured search strings were applied to retrieve highly relevant documents (see Supplementary Table S1 for full details).

This combined bibliometric–systematic review approach was chosen because it provides distinct advantages over traditional narrative or purely systematic reviews. Bibliometric analysis provides an objective, data-driven map of research trends, influential contributors, and conceptual networks, while systematic reviews offer deeper thematic and theoretical insights. Together, these methods enhance the rigour and validity of findings by triangulating quantitative mapping with qualitative synthesis, yielding a comprehensive understanding of how AI has been used to scaffold metacognition in STEM education.

### 2.2. Inclusion and Exclusion Criteria

To ensure relevance and rigor, documents were included based on the following criteria: (1) published between 2005 and 2025, (2) written in English, (3) peer-reviewed journal articles or conference proceedings, (4) focused on metacognitive development in STEM education or broader education and learning contexts of AI integration, and (5) explicitly referenced theoretical or conceptual frameworks. Documents were excluded if they were (a) not in English, (b) editorials, reviews, or book chapters, (c) not directly related to both AI and metacognition, or (d) lacked educational context. We selected 2005 as the starting point because this period marks the emergence and wider adoption of AI-based tools in education, including intelligent tutoring systems, adaptive learning platforms, and early learning analytics applications (e.g., Desmarais and Baker 2012). Furthermore, bibliometric scoping revealed that publications addressing AI and metacognition in STEM education began to grow substantially after 2005, making it a meaningful cutoff for capturing contemporary developments.

The database search yielded 168 records, of which 33 duplicates were removed, leaving 135 unique studies for screening. Ninety-five were excluded at the title/abstract stage, and 40 full-text articles were assessed for eligibility. Sixteen full-texts were excluded—6 did not address AI and metacognition jointly, 4 were not STEM-related, 3 were non-empirical, and 3 for other reasons—resulting in 24 studies included in the systematic review.

### 2.3. Data Retrieval and Screening Reliability

A total of 119 documents were retrieved from Scopus and 49 from Web of Science. Following deduplication and file merging in RStudio (version 2025.05.1+513), a final dataset of 135 unique records was established for bibliometric analysis. These documents were saved in BibTeX and CSV formats for compatibility with VOSviewer (version 1.6.20) and the Biblioshiny interface in RStudio. Following retrieval and cleaning, the included studies were thematically coded. Thematic codes were developed through an iterative review process. Two researchers independently coded an initial subset of 10 studies to refine the codebook before applying it to the remaining articles. To ensure screening rigour, both reviewers independently evaluated all titles, abstracts, and full texts against the inclusion/exclusion criteria. Agreement was assessed using Cohen’s κ, showing substantial reliability (κ = 0.82 for title/abstract screening; κ = 0.87 for full-text). Discrepancies were resolved through discussion with a third reviewer. The final thematic categories are summarised in Table 1.

### 2.4. Bibliometric Analysis

Using VOSviewer and Biblioshiny, the study generated co-occurrence maps, keyword clustering, author collaboration networks, and thematic evolution diagrams. This helped to identify key themes, highly cited works, and emerging concepts in the literature related to AI, metacognition, and STEM education (Aria and Cuccurullo 2017).

### 2.5. Systematic Review and PRISMA Approach

**Figure 1:**
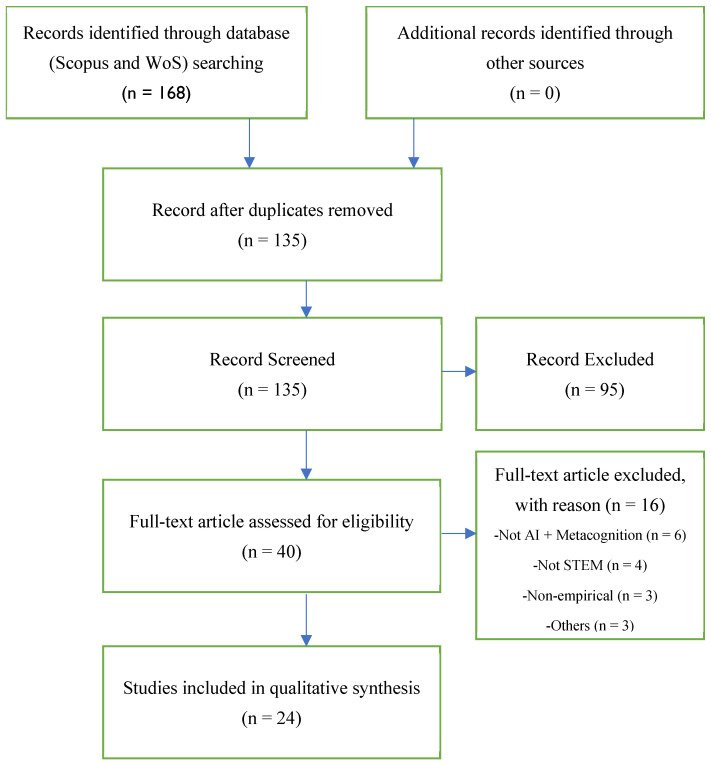
PRISMA 2020 flow diagram summarising the study-selection process. *Note.* This figure illustrates the systematic process used for identifying and screening, and includes studies that examined the facilitation of metacognition in AI-supported STEM classrooms.

Following PRISMA 2020 guidelines (Page et al. 2021), a subset of the 135 bibliometric records was systematically screened through the four phases—Identification, Screening, Eligibility, and Inclusion—to identify the 24 studies selected for qualitative synthesis (see Figure 1).

These studies were analysed to explore how metacognitive strategies are integrated with AI tools in STEM education contexts. The review went beyond theoretical inquiry, capturing empirical insights on implementing such combinations in classroom practice. Key data points extracted from each study included: the theoretical or conceptual frameworks employed, the nature and purpose of metacognitive strategies used, the specific AI tools or systems applied (e.g., intelligent tutoring systems, chatbots, adaptive platforms), the STEM discipline and educational level (e.g., primary, secondary or tertiary), and the study’s alignment with human-centred or posthumanist paradigms (see Table 1 for the empirical studies integrating AI and metacognition in STEM education). In clarifying the posthumanist paradigm, we note that what is “shared” is not metacognition itself, but rather the regulation of learning experiences, where metacognitive processes are scaffolded by AI tools alongside human agency. The data were coded thematically using a structured Excel matrix, which enabled analysis of trends in theoretical orientation, the evolving relationship between AI and metacognition, and the pedagogical implications of these interactions across STEM learning environments.

Across the reviewed studies, AI applications supporting metacognition were most implemented in mathematics and science education, particularly in secondary and higher education contexts. Intelligent Tutoring Systems, learning analytics dashboards, and adaptive learning platforms were the most prevalent AI tools used to scaffold metacognitive regulation and awareness. Despite this progress, significant research gaps persist across STEM domains and various educational levels. Primary-school and early-childhood STEM education are scarcely represented, and few studies examine AI-driven metacognitive interventions in teacher-education or pre-service training. Similarly, engineering and technology subjects receive far less attention than mathematics and science. These imbalances suggest that current research is concentrated in only two of the four STEM areas, indicating the need for broader, domain-inclusive exploration of AI-mediated metacognition.

### 2.6. Summary of Findings

Analysis of the 24 reviewed studies reveals a clear dominance of human-centred approaches, with 23 studies explicitly framed within the context of human agency. In contrast, only one study (Walker et al. 2025) reflected a posthumanist orientation. Most interventions focused on planning, monitoring, and reflection strategies, particularly within mathematics and general STEM contexts. Secondary and tertiary education levels were the most represented, while primary-level studies remained underrepresented. These findings indicate that while AI tools are increasingly used to scaffold metacognition, theoretical perspectives remain largely traditional, highlighting an important gap for future exploration of hybrid or posthumanist models.

## 3. Results

The findings are organised and reported according to the structure of the research questions guiding this study.

### 3.1. RQ1: What Are the Publication Trends on Metacognition in STEM Education Between 2005 and 2025?

**Figure 2:**
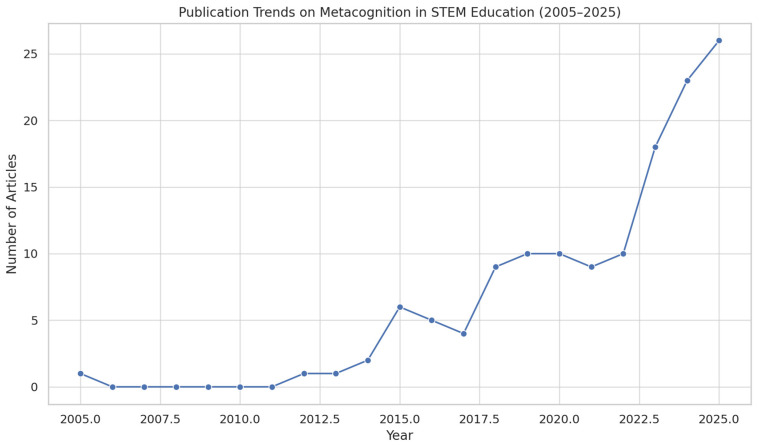
Annual Publication Trends on Metacognition in STEM Education (2005–2025). *Note.* This figure displays the number of publications per year from 2005 to 2025 related to metacognition in STEM education. Data were retrieved from a bibliometric analysis of 135 documents using Bibliometrix and Biblioshiny.

The publication output on metacognition in STEM education has steadily increased since 2005, with notable growth after 2015 (see Figure 2). The annual output grew significantly from a single in 2005, indicating a rising scholarly interest. The trend suggests a peak in recent years, which aligns with global attention on educational innovation, artificial intelligence, and learner autonomy.

### 3.2. RQ2: What Are the Most Frequently Occurring AI-Related Concepts and Tools in the Literature on Metacognition in STEM Education?

**Figure 3:**
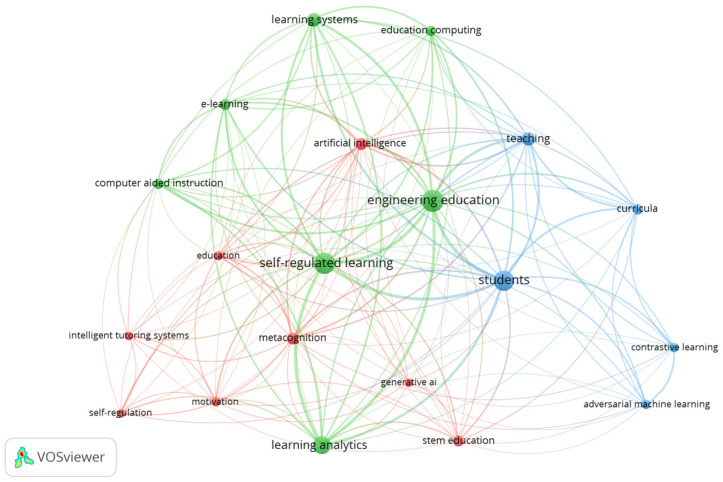
Keyword co-occurrence network map of AI-related terms. *Note.* This map was generated using VOSviewer based on a minimum occurrence threshold of 10. Node size indicates keyword frequency. Line thickness shows the strength of co-occurrence links between keywords.

A keyword co-occurrence analysis was conducted to identify the most prominent AI-related concepts and tools within the literature on metacognition in STEM education. As shown in Table 2 (ranked by frequency) and visualised in Figure 3, the most frequently occurring AI-related term was *learning analytics* (n = 43), underscoring its centrality in supporting and monitoring self-regulated learning processes. Other high-ranking terms include *learning systems* (n = 25), *artificial intelligence* (n = 20), and *e-learning* (n = 17), suggesting widespread integration of digital and intelligent technologies in STEM education. Notably, emerging approaches such as *adversarial machine learning* and *contrastive learning* (each n = 11), along with *generative AI* (n = 10), reflect increasing interest in advanced AI models. Traditional applications, such as intelligent tutoring systems (n = 10), also remain relevant, particularly in fostering the use of metacognitive strategies. These findings illustrate a shift from established AI tools toward more sophisticated, data-driven educational technologies.

### 3.3. RQ3: Which Journals, Authors, and Countries Contribute Most to the Literature on AI and Metacognition in STEM Education?

#### 3.3.1. Journal Contributions to Research on AI and Metacognition in STEM Education

A source analysis was conducted to identify the key publication venues contributing to research on AI and metacognition in STEM education. Results show that the literature is published in peer-reviewed journals and major conference proceedings. As presented in Table 3, the *Lecture Notes in Computer Science (LNCS)* series led with 16 publications, reflecting strong contributions from computer science-driven education research. This is followed by *British Journal of Educational Technology*, *Education and Information Technologies*, and *Frontiers in Education*, each with five articles. Notable venues, such as Computers & Education, IEEE EDUCON, and the Frontiers in Education Conference (FIE), contributed four articles each, highlighting sustained academic and practitioner interest. Furthermore, journals such as Computer Applications in Engineering Education, Computers in Human Behaviour, and Educational Psychology Review each produced three articles, signalling a growing interdisciplinary engagement with AI-supported metacognitive research.

#### 3.3.2. Author Contributions to Research on AI and Metacognition in STEM Education

**Figure 4:**
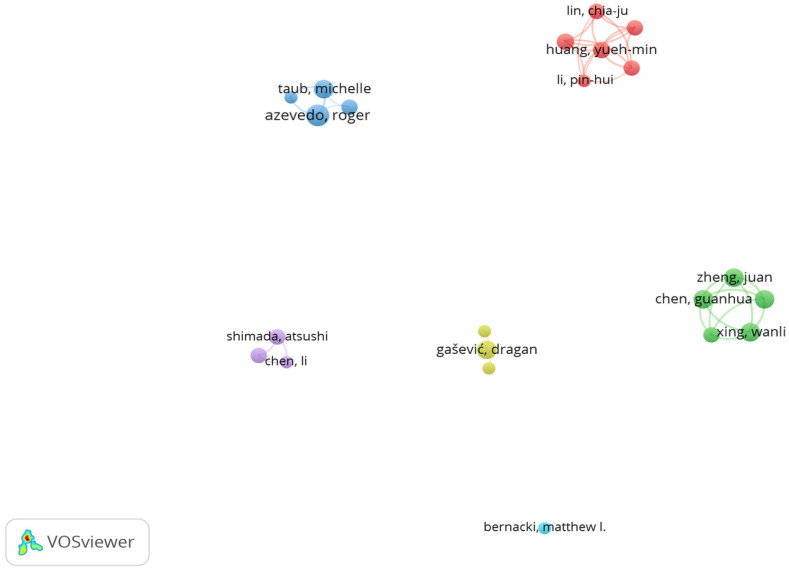
Author Co-authorship Network on AI and Metacognition in STEM Education. *Note*. The network shows co-authorship patterns among the most productive authors in the field.

A co-authorship and productivity analysis revealed leading contributors to the literature on AI and metacognition in STEM education. As shown in Table 4 and visualised in Figure 4, *Roger Azevedo* leads with eight publications and 399 citations, reflecting strong and sustained engagement. *Dragan Gašević* follows with six publications and the highest citation count (720), highlighting his significant influence in the field. Other frequently publishing authors include *Guanhua Chen*, *Charles Xie*, *Wanli Xing*, *Juan Zheng*, and *Michelle Taub*, each with six publications. The VOSviewer map in Figure 4 displays author co-authorship networks, where the largest connected cluster comprises six items, indicating limited but growing collaboration among core researchers. These findings suggest a concentrated yet expanding scholarly community contributing to AI-driven metacognitive research in STEM education.

#### 3.3.3. Country Contributions to Research on AI and Metacognition in STEM Education

**Figure 5:**
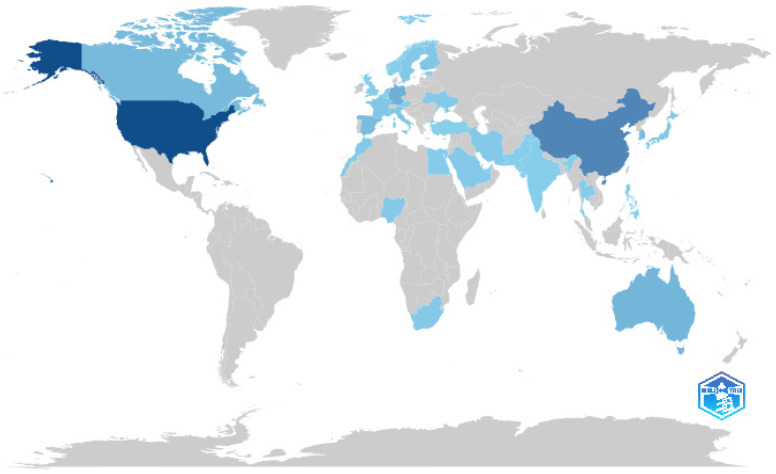
Country Scientific Production on AI and Metacognition in STEM Education. *Note*. Visualisation generated using Biblioshiny. The map illustrates the geographic distribution of publication volume by country, based on corresponding author affiliations.

A bibliometric analysis of 135 publications, as shown in Table 5, revealed that the United States is the leading contributor to research on AI and metacognition in STEM education, with 68 publications, followed by China (37), Germany (16), Australia (13), and Canada (11). Other active contributors include South Korea and Spain (10 each), while Finland, France, and Italy each produced five publications. These results, visualised in Figure 5, illustrate the global distribution of scientific productivity in this field.

### 3.4. RQ4: Which Theoretical Frameworks Are Most Frequently Associated with Studies on AI and Metacognition in STEM, and How Have These Evolved?

Across the 25 reviewed studies, as shown in Table 1, Self-Regulated Learning (SRL) Theory is the most frequently used framework, particularly between 2010 and 2020. Zimmerman’s model of SRL, focused on planning, monitoring, and reflection, underpins many studies involving AI tools like dashboards and adaptive systems (e.g., Ader 2019; Braad et al. 2022). From 2017 onward, the literature shows greater theoretical diversity. Frameworks such as Socially Shared Metacognition (Lobczowski et al. 2021), Design-Based Research (Baten et al. 2017), and Reflective Pedagogy (Siegesmund 2017) are responsive to more collaborative and technology-mediated environments. Recent models, including ALERT and AI Safety Design (Willison et al. 2023; Walker et al. 2025), signal a shift toward system-level metacognition embedded in AI. This trend reflects a move from individual learner regulation toward co-regulation within human–AI systems, expanding theoretical focus to include design ethics and technological cognition (Table 1, Columns 8–9).

### 3.5. RQ5: How Have Keywords and Conceptual Language in the Literature Shifted from Human-Centred to Posthumanist Paradigms in the Context of AI and Metacognition?

A conceptual shift is equally observable in the keywords and ontologies used across the studies. Earlier research (e.g., Kaplan 2008; Pennequin et al. 2010) emphasised human-centred constructs such as reflection, self-awareness, regulation, and teacher scaffolding. As shown in Table 1, Column 9, the discourse remained embedded within the learner’s cognitive boundaries and relied heavily on the teacher or instructional design as the primary agent orchestrating metacognitive growth. In contrast, more recent studies (e.g., Walker et al. 2025) reflect an emerging posthumanist orientation, where metacognition is enacted by humans and embedded within or distributed across technological systems. Here, metacognition is no longer exclusively a human trait but a designable and operational feature of AI systems. Terms such as system-level reflection, machine awareness, AI safety frameworks, and autonomous decision regulation suggest that agency is increasingly diffused across networks of human and non-human actors.

This evolution marks a significant paradigm shift: from instrumental AI serving human learning to entangled learning ecologies in which humans and machines co-participate in reflective and self-regulatory processes. This shift transforms the learner–AI relationship and challenges conventional boundaries of cognition, strategy, and educational agency in STEM contexts.

## 4. Discussion

This section interprets the key findings thematically, providing possible explanations that are supported by relevant theories and literature. It examines the evolution of research on AI and metacognition in STEM, highlighting trends, tools, contributors, theoretical shifts, and emerging paradigms.

### 4.1. Growing Research Attention on AI and Metacognition in STEM Education

The steady rise in research output on metacognition within STEM education, especially after 2015, may be explained by increasing global attention to learner autonomy, personalised education, and the digital transformation of classrooms. As AI technologies have become more accessible and widely adopted in education systems, scholars have naturally been drawn to investigate how these tools affect higher-order learning processes, such as metacognition. Furthermore, the widespread emphasis on 21st-century skills, such as critical thinking, problem-solving, and self-regulated learning, likely contributed to this surge in interest. Researchers have sought to understand not only how students learn, but also how they learn to learn, particularly within the complex and rapidly evolving contexts of science and mathematics. This pattern aligns well with Flavell’s (1979) Theory of Metacognition, which posits that metacognition is essential for effective learning. The increasing research interest reflects recognition of the importance of metacognitive knowledge and regulation (planning, monitoring, and evaluation) in enabling students to succeed in cognitively demanding STEM environments.

Furthermore, the systemic nature of this research growth resonates with GST, as it illustrates how evolving technological, pedagogical, and psychological subsystems collectively shape educational research trajectories (Von Bertalanffy 1950). The literature supports this trend. Studies have consistently shown that metacognitive strategies improve learning outcomes in mathematics and science (e.g., Zepeda et al. 2019; Clark et al. 2025; Smith and Mancy 2018). These findings justify the intensifying research interest and affirm that metacognition has become central in AI-permeated STEM education as a theoretical construct and instructional target.

### 4.2. Learning Analytics and AI Tools as Scaffolds for Metacognitive Regulation

The prominence of terms like *learning analytics*, *learning systems*, and *intelligent tutoring systems* suggests that much of the research on AI and metacognition in STEM education has focused on how digital tools can support learners in planning, monitoring, and evaluating their learning. This focus likely stems from the growing demand for scalable, data-driven approaches to support self-regulated learning. As classrooms become increasingly digitised and complex, educators and learners require systems that offer timely feedback, track cognitive engagement, and guide real-time decision-making processes. These tools are not just replacing traditional instruction; they act as cognitive partners that help externalise metacognitive processes.

This development aligns conceptually with the GST, emphasising the interdependence of components in complex systems (Von Bertalanffy 1950). AI tools are subsystems that interact with instructional design, learner behaviour, and cognitive demands to create adaptive learning environments. These environments promote metacognitive growth by dynamically responding to learner inputs, just as a well-functioning system adapts to maintain equilibrium. In this context, learning regulation and learning experiences, supported through metacognitive scaffolds, become a shared responsibility between human and machine components. This trend is well-supported in the literature. For example, Roll and Winne (2015) showed how analytics tools embedded in online learning platforms improved students’ metacognitive awareness by visualising their behaviours and providing reflective feedback. Similarly, Holstein and Aleven (2021) found that tools like Lumilo and smart glasses enhanced teachers’ and students’ abilities to monitor and regulate learning in real time. These studies confirm that AI-powered systems can scaffold key metacognitive functions, especially in STEM classrooms where abstract reasoning and problem-solving are central. The shift toward digital metacognitive support thus reflects both technological capability and pedagogical necessity.

### 4.3. Scholarly Ecosystem—Productive Journals, Authors, and Countries

The dominance of certain journals, authors, and countries in this field highlights the increasingly interdisciplinary and globally coordinated nature of research at the intersection of AI, metacognition, and STEM education. The leading role of venues like *Lecture Notes in Computer Science (LNCS)* and the *British Journal of Educational Technology* suggests that educational researchers and computer scientists are shaping the field. This may reflect a convergence of priorities: educators seek technological supports for metacognitive development, while technologists embed cognitive theories into intelligent systems. The strong presence of engineering and psychology journals further underscores the multidisciplinary character of the field.

Prominent scholars such as *Roger Azevedo* and *Dragan Gašević*, known for their work on self-regulated learning and learning analytics, contribute heavily to this discourse. Their sustained engagement reflects a maturing field in which theoretical development is closely tied to technological innovation. Countries like the *United States* and *China* dominate the landscape, likely due to their greater access to funding, infrastructure, and institutional support for integrating AI in education. The rise of authors from these countries also suggests that global leadership in educational AI research is deeply linked to national investments in AI development and STEM policy priorities. For example, in the United States, initiatives such as the National AI Research Institutes (established by the National Science Foundation in 2020) and the Federal STEM Education Strategic Plan (2018) have directed substantial funding toward integrating AI and STEM in education. Similarly, in China, the 2017 Next Generation Artificial Intelligence Development Plan explicitly prioritised AI in education, with national-level funding allocated to intelligent tutoring systems, adaptive learning platforms, and AI-supported STEM curricula. These sustained investments help explain the concentration of research leadership in these two countries and provide context for their dominant scholarly contributions.

This distributed scholarly activity is well-explained by GST, which frames educational research as a complex system shaped by interacting subsystems, including academic publishing ecosystems, funding environments, and disciplinary networks. Research clustering in particular regions and institutions reflects system-level feedback loops, where resource availability, research visibility, and citation impact reinforce continued output. These findings also align with trends in the literature. For instance, Clark et al. (2025) and Fatimah et al. (2024) note a sharp rise in AI-STEM scholarship over the last decade, particularly in technologically advanced regions. The field’s evolution is thus not only conceptual but also structurally driven by collaborations among productive scholars, high-impact journals, and innovation-focused countries. While this concentration suggests a strong knowledge base, it also points to the need for broader global engagement to ensure the inclusive development of AI tools and metacognitive frameworks across diverse STEM contexts.

### 4.4. Theoretical Evolution—From Individual Regulation to System-Level Metacognition

Theoretical frameworks used in the reviewed literature demonstrate a clear progression from traditional models of individual metacognitive control to more complex, distributed frameworks that reflect the evolving nature of learning environments. The early dominance of Self-Regulated Learning theory, particularly Zimmerman’s model, can be attributed to its strong alignment with foundational metacognitive processes, planning, monitoring, and evaluating, especially in the context of student autonomy (e.g., Ader 2019; Braad et al. 2022). As AI technologies began to mediate more aspects of learning, researchers increasingly incorporated complementary frameworks, such as Socially Shared Metacognition (Lobczowski et al. 2021), Reflective Pedagogy (Siegesmund 2017), and Design-Based Research (Baten et al. 2017), to account for the co-regulatory and collaborative dimensions of learning. This evolution resonates with General Systems Theory, which views learning environments as dynamic networks of human and technological actors (Von Bertalanffy 1950, 1968). More recent work in complexity science (Mitchell 2009) strengthens this interpretation by emphasising adaptive interactions within complex systems; the emergence of system-level metacognition can be understood as one such adaptive phenomenon. Only one reviewed study (Walker et al. 2025) explicitly adopted a posthumanist orientation, embedding metacognitive reflection at the system level within AI design. While this single case does not signal a widespread paradigm shift, it represents an important departure from human-centred assumptions. When viewed alongside the bibliometric evidence—such as emerging keywords like system-level reflection and machine awareness—this suggests the beginnings of a theoretical diversification that may open space for posthumanist framings in the future.

### 4.5. Conceptual Shifts from Human-Centred to Posthumanist Frames

The conceptual language across the corpus reveals both continuity and subtle change. Earlier studies emphasised human-centred constructs such as reflection, self-awareness, and teacher scaffolding (e.g., Kaplan 2008; Pennequin et al. 2010; Lobczowski et al. 2021), reinforcing the assumption that cognition resides solely within the learner. More recent contributions, though still few in number, introduce terms such as *autonomous decision regulation* and *AI safety frameworks*, which imply a broader distribution of agency across human and technological actors. Although the systematic review shows that explicit posthumanist framings are rare (1 of 24 studies; see Walker et al. 2025), these linguistic and conceptual signals in the broader bibliometric dataset are consistent with the posthumanist perspectives discussed by scholars such as Cukurova (2024) and Lim 2025. Together, they suggest a nascent posthumanist discourse. It would therefore be premature to describe a consolidated “trend,” but it is reasonable to note that posthumanist perspectives are emerging as an alternative orientation. This interpretation recognises the dominance of human-centred paradigms while also acknowledging the potential for hybrid human–AI framings to shape future theorisation and practice in STEM education.

### 4.6. Clarifying “Scaffolding” Versus “Sharing” Metacognition

Building on this shift toward posthumanist framing, it is necessary to clarify how AI’s role in supporting metacognition is conceptualised, particularly in distinguishing scaffolding from sharing. We recognise an important distinction between scaffolding and sharing metacognition. *Scaffolding* treats metacognition as a human capacity that can be supported and extended through prompts, feedback, or adaptive interventions, thereby allowing learners to retain agency and responsibility. *Sharing*, by contrast, distributes metacognitive functions between human and AI agents, where AI may compensate for weak regulation (e.g., automating monitoring or evaluation). While this reflects posthumanist views on hybrid intelligence, it risks limiting learners’ opportunities to develop independent strategies if applied uncritically. Both perspectives acknowledge AI’s active role, but their implications diverge: scaffolding supports gradual learner growth, whereas sharing risks substitution. Our review positions AI primarily as a scaffolding tool for metacognition to strengthen learners’ reflective capacities, while noting that sharing must be critically evaluated.

## 5. Conclusions

This study contributes to the understanding of how artificial intelligence (AI) tools intersect with metacognition in STEM education by combining bibliometric and systematic review approaches. The analysis demonstrates that publications in this field have increased steadily, with a growing shift toward integrating AI-supported systems such as intelligent tutoring, adaptive feedback, and learning analytics into instructional design. Most of the reviewed studies focused on mathematics and science education, reflecting the dominance of these disciplines in AI-assisted metacognitive research. Conceptually, the review identified that human-centred paradigms continue to guide most AI-metacognition studies. At the same time, only a few papers adopt posthumanist or hybrid perspectives that view AI as a cognitive partner rather than a tool. Despite these advances, methodological, theoretical, and ethical limitations remain. Few studies employed longitudinal or experimental research designs capable of identifying causal or sustained effects of AI-mediated metacognitive interventions. Research also remains uneven across STEM domains, with limited representation in technology and engineering education as well as in early-childhood and teacher-education contexts. Ethical considerations concerning data privacy, transparency, and learner agency are often underexplored, underscoring the need for guiding frameworks that balance innovation with responsibility.

In conclusion, this review proposes four specific recommendations for advancing the field. First, longitudinal and experimental studies should be prioritised to examine the long-term effects of AI-supported metacognitive interventions. Second, hybrid and posthumanist frameworks should be expanded to understand how human and machine cognition co-construct learning. Third, the development of explicit ethical frameworks is required to ensure transparency, fairness, and accountability in AI-metacognitive systems. Ultimately, future research should extend to underrepresented STEM domains—particularly technology, engineering, and early childhood education—to promote inclusivity and equity in AI-mediated learning research. Collectively, these directions outline a balanced agenda for strengthening the theoretical, methodological, and ethical foundations of AI-metacognition research in STEM education.

## Figures and Tables

**Table 1:** Summary of Empirical Studies Integrating AI and Metacognition in STEM Education.

S/N	Author(s) & Year	Country	Focus of Study	Level	Metacognitive Strategy Used	AI Tool Applied	STEM Discipline	Theoretical Framework	Human/Posthuman Orientation	Key Findings/Notes
1	Bogdanović et al. (2022)	Serbia	Relationship between physics performance and metacognition among elementary students	Primary	Planning, regulation, evaluation	Simulation software	Physics	Cognitive Load Theory	Human	Strong correlation between metacognition and achievement
2	Zhang and Li (2021)	China	Integrating active learning and metacognition into STEM writing	Tertiary	Reflective writing strategies	Data analytics tools	Biology/Physiology	Constructivist pedagogy	Human	Metacognitive strategies improved writing outcomes in science disciplines
3	Ader (2019)	France	Teacher support for SRL in math	Secondary	SRL strategy use	Learning analytics platforms	Mathematics	SRL Theory	Human	Teacher involvement is key to metacognitive development
4	Willison et al. (2023)	Australia	Use of the ALERT model for building metacognition in science	Tertiary	ALERT learning cycle	AI-based learning systems	Science (general)	ALERT Framework	Human	Students improved in reflective thinking and independent inquiry
5	Baten et al. (2017)	Belgium	Role of metacognition in instructional design	Secondary	Monitoring, evaluation	Adaptive learning systems	Mathematics	Design-Based Research	Human	Instructional design improved learning through metacognitive scaffolding
6	McCord and Matusovich (2019)	USA	Engineering students’ metacognitive engagement	Tertiary	Metacognitive engagement strategies	Online SRL platforms	Engineering	SRL Theory	Human	Increased awareness of strategies led to academic gains
7	Fung and Poon (2021)	Hong Kong	Dynamic activities and metacognitive learning in math	Secondary	Reflective thinking	Interactive simulations	Mathematics	Constructivist approach	Human	Tools enhanced student conceptual understanding and metacognitive control
8	Braad et al. (2022)	Sweden	Enhancing SRL and metacognition through digital tools	Tertiary	SRL support	SRL dashboard tools	STEM (general)	Zimmerman’s SRL Framework	Human	Learners showed more goal-setting and strategic learning behaviours
9	Lobczowski et al. (2021)	USA	Shared metacognition in STEM project-based learning	Secondary	Socially shared metacognition	Project-based AI analytics	STEM (general)	Social Constructivism	Human	Peer dialogue promoted joint regulation and awareness
10	Siegel (2012)	USA	Group metacognitive dialogue during science problem-solving	Secondary	Collaborative regulation	Computer simulations	Science	Situated cognition	Human	Groups regulated thought through shared metacognitive practices
11	Swanson et al. (2024)	USA	Impact of structured interventions on academic metacognition	Tertiary	Academic metacognitive strategies	Dashboard analytics	STEM (general)	Experimental pedagogy	Human	Structured prompts increased students’ metacognitive use
12	Zhao et al. (2019)	China	Linking metacognition and math problem-solving performance	Secondary	Strategic planning and reflection	Data analysis software	Mathematics	Cognitive-metacognitive model	Human	Strong predictors of math success were metacognitive strategy use
13	Branigan and Donaldson (2019)	UK	Role of teacher-student interaction in metacognition	Primary	Teacher-mediated reflection	Interactive whiteboards	General STEM	Sociocultural theory	Human	Effective metacognitive dialogue fostered deeper thinking
14	Kaplan (2008)	USA	Clarifying SRL and metacognition frameworks	Tertiary	Framework analysis	Not specific	STEM (general)	SRL Theory	Human	Provided theoretical clarity and integration of metacognition with SRL
15	Aghabeygi and Khanjani (2020)	Iran	Training program on metacognitive skills	Tertiary	Explicit instruction	Online learning systems	Health Sciences	Constructivist instructional design	Human	Training improved academic performance and learner motivation
16	Oppong et al. (2019)	Ghana	Gifted learners’ metacognitive strategy use	Secondary	SRL and reflection	Classroom analytics	STEM (general)	Gifted Education theory	Human	Gifted learners utilised metacognitive strategies more frequently
17	Hidayat et al. (2022)	Indonesia	Influence of metacognition on math scores	Secondary	Self-monitoring, evaluation	Online quiz systems	Mathematics	Metacognitive awareness theory	Human	Higher awareness correlated with better performance
18	Roque Herrera et al. (2025)	Chile	Metacognitive awareness among health science students	Tertiary	Metacognitive awareness inventory	LMS platforms	Health Sciences	Flavell’s Metacognition Theory	Human	Recommended embedding of metacognitive training in curricula
19	Medina et al. (2017)	USA	Strategies to improve metacognition in pharmacy education	Tertiary	Reflective questioning	E-learning platforms	Pharmacy	Reflective practice model	Human	Learners benefited from feedback and structured self-assessment
20	Pennequin et al. (2010)	France	Effect of metacognitive training in adult math learners	Tertiary	Training in regulation and planning	Computer-based tools	Mathematics	Cognitive strategy instruction	Human	Adult learners developed better strategies post-training
21	Walker et al. (2025)	USA	Metacognition in AI safety design	Tertiary	System-based reflection	AI models	Computer Science	AI safety and design framework	Posthuman	Reframes metacognition as design architecture for responsible AI
22	Shields et al. (2024)	USA	Role of help-seeking and reflection in science metacognition	Primary	Reflection and help-seeking	Tablet-based science tools	Science	Metacognitive development theory	Human	Tools supported young learners’ ability to self-regulate and seek help
23	Siegesmund (2017)	USA	Developing metacognitive learners in microbiology	Tertiary	Reflective practice	Online learning platforms	Biology	Reflective pedagogy	Human	Emphasis on student reflection enhanced long-term learning
24	Jeong and Kim (2025)	South Korea	Encouraging metacognitive questioning in science class	Secondary	Metacognitive inquiry	Automated feedback tools	Science	Inquiry-based learning framework	Human	Metacognitive questioning improved students’ scientific problem-solving

*Note*. Of the 24 reviewed studies, 23 were coded as human-centred and only 1 (Walker et al. 2025) was explicitly posthumanist. Coding reflects the theoretical orientation stated by the authors of each study.

**Table 2:** Frequently Occurring AI-Related Concepts and Tools in Literature on Metacognition in STEM Education.

Rank	AI-Related Keyword	Occurrences
1	Learning analytics	43
2	Learning systems	25
3	Artificial intelligence	20
4	e-learning	17
5	Computer-aided instruction	15
5	Education computing	15
7	Adversarial machine learning	11
7	Contrastive learning	11
9	Generative AI	10
9	Intelligent tutoring systems	10

**Table 3:** Top 10 Publication Venues Contributing to Research on AI and Metacognition in STEM Education.

Rank	Journal Title	Articles
1	Lecture Notes in Computer Science (LNCS)	16
2	British Journal of Educational Technology	5
2	Education and Information Technologies	5
2	Frontiers in Education	5
5	Computers & Education	4
5	IEEE Global Engineering Education Conference, EDUCON	4
5	Proceedings—Frontiers in Education Conference, FIE	4
8	Computer Applications in Engineering Education	3
8	Computers in Human Behaviour	3
8	Educational Psychology Review	3

**Table 4:** Top Contributing Authors to Research on AI and Metacognition in STEM Education.

Rank	Author Name	Documents	Citations
1	Azevedo, Roger	8	399
2	Gasevic, Dragan	6	720
3	Chen, Guanhua	6	360
3	Xie, Charles	6	360
3	Xing, Wanli	6	360
3	Zheng, Juan	6	360
7	Taub, Michelle	6	243
8	Huang, Yuen-Min	5	32
8	Lee, Hsin-Yu	5	32
10	Li, Shan	4	193

*Note.* Ranking is based primarily on the number of publications and total citations, with the latter used for tie-breaking.

**Table 5:** Top 10 Countries Contributing to Research on AI and Metacognition in STEM Education.

Rank	Country	Articles
1	USA	68
2	China	37
3	Germany	16
4	Australia	13
5	Canada	11
6	South Korea	10
6	Spain	10
8	Finland	5
8	France	5
8	Italy	5

*Note.* Counts reflect the number of publications per country from 2005 to 2025.

## Data Availability

No new data were created or analysed in this study.

## Supplementary Materials

The following supporting information can be downloaded at: https://www.mdpi.com/article/10.3390/jintelligence13110148/s1. Table S1. Boolean Search Strings for Scopus and Web of Science Used in the Bibliometric–Systematic Review.

## Abbreviations

The following abbreviations are used in this manuscript:

AI	Artificial Intelligence
STEM	Science, Technology, Engineering and Mathematics
PRISMA	Preferred Reporting Items for Systematic Reviews and Meta-Analyses
ITS	Intelligent Tutoring Systems
GST	General Systems Theory
RQ	Research Question(s)
