# Supplementary material for: Mapping the Scaffolding of Metacognition and Learning by AI Tools in STEM Classrooms: A Bibliometric–Systematic Review Approach (2005–2025)
Source: J Intell. 2025 Nov 15;13(11):148. doi: 10.3390/jintelligence13110148 (PMC12653222; doi:10.3390/jintelligence13110148)
Supplement: Supplementary file 1 [file jintelligence-13-00148-s001.zip › jintelligence-3876850-Supplementary Material.pdf]

Supplementary Materials

Table S1. Boolean Search Strings for Scopus and Web of Science Used in the Bibliometric–Systematic Review

Database	Search String
**Scopus**	*(TITLE-ABS-KEY("metacognition" OR "metacognitive strategies" OR "metacognitive awareness" OR "metacognitive skills" OR "self-regulated learning" OR "reflective thinking" OR "learning regulation") AND TITLE-ABS-KEY("artificial intelligence" OR "AI tools" OR "machine learning" OR "generative AI" OR "chatbots" OR "ChatGPT" OR "intelligent tutoring systems" OR "adaptive learning systems" OR "learning analytics" OR "AI-powered feedback" OR "natural language processing" OR "robotics in education") AND TITLE-ABS-KEY("STEM education" OR "science education" OR "technology education" OR "engineering education" OR "mathematics education" OR "physics education" OR "chemistry education" OR "biology education" OR "STEM classrooms" OR "STEM learning") AND PUBYEAR > 2004 AND PUBYEAR < 2026 AND (DOCTYPE(ar) OR DOCTYPE(cp)) AND (LIMIT-TO(LANGUAGE, "English")))*
**Web of Science**	*((ALL=("metacognition" OR "metacognitive strategies" OR "metacognitive awareness" OR "metacognitive skills" OR "self-regulated learning" OR "reflective thinking" OR "learning regulation")) AND ALL=("artificial intelligence" OR "AI tools" OR "machine learning" OR "generative AI" OR "chatbots" OR "ChatGPT" OR "intelligent tutoring systems" OR "adaptive learning systems" OR "learning analytics" OR "AI-powered feedback" OR "natural language processing" OR "robotics in education")) AND ALL=("STEM education" OR "science education" OR "technology education" OR "engineering education" OR "mathematics education" OR "physics education" OR "chemistry education" OR "biology education" OR "STEM classrooms" OR "STEM learning"))*
